# The role of *Fusobacterium nucleatum* in macrophage M2 polarization and NF-κB pathway activation in colorectal cancer

**DOI:** 10.3389/fimmu.2025.1549564

**Published:** 2025-04-03

**Authors:** Wei Zheng, Yuxin Wang, Haoyang Sun, Surina Bao, Shuai Ge, Chunshan Quan

**Affiliations:** ^1^ Key Laboratory of Biotechnology and Bioresources Utilization of Ministry of Education, College of Life Science, Dalian Minzu University, Dalian, China; ^2^ Department of Bioengineering, College of Life Science, Dalian Minzu University, Dalian, Liaoning, China

**Keywords:** *Fusobacterium nucleatum*, colorectal cancer, macrophage polarization, NF-κB signaling pathway, trascriptome

## Abstract

*Fusobacterium nucleatum* is strongly linked to colorectal cancer (CRC) progression, but its mechanisms for influencing macrophage polarization and tumor development are not well understood. We established an *in vitro* model of *F. nucleatum* infection in RAW264.7 macrophages to investigate these processes. Macrophage polarization was evaluated using scanning electron microscopy (SEM), real-time quantitative PCR (RT-qPCR), and immunofluorescence staining. RNA sequencing (RNA-Seq) identified differentially expressed genes (DEGs) and enriched pathways, focusing on the role of the NF-κB signaling pathway in macrophage polarization. *F. nucleatum* infection induced M2 polarization in RAW264.7 macrophages, as confirmed by SEM analysis and RT-qPCR validation. A total of 2,029 DEGs were identified after *F. nucleatum* infection, with 763 upregulated and 1,266 downregulated. GO and KEGG enrichment analysis showed that cytokine-cytokine receptor interaction, TNF signaling, and NF-κB signaling pathways are upregulated in macrophages after *F. nucleatum* infection, indicating enhanced cytokine activity and immune response. Key genes (*Nfkb1*, *Nfkb2*, *Malt*, *Lta*, *Ltb*, *Tnf*) and proteins (P50, P100) in the NF-κB pathway are upregulated, indicating the crucial role of the NF-κB pathway in M2 macrophage polarization. This study offers crucial evidence regarding the role of the NF-κB signaling pathway in modulating *F. nucleatum*-induced macrophage M2 polarization, underscoring its significance in the progression of colorectal cancer.

## 1 Introduction

Colorectal cancer (CRC) is one of the most common malignancies globally and poses a significant threat to human health (1–3). Recent accumulating evidence has shown that the human gut bacterium *Fusobacterium nucleatum* is associated with the progression of colorectal cancer (4, 5). *F. nucleatum* influences all stages of CRC development by creating a pro-inflammatory microenvironment and promoting CRC cell proliferation and migration. (6–9). Research has shown that *F. nucleatum*, which is enriched in CRC tissues, can induce oncogenic inflammatory responses and impair anti-tumor immunity by expressing the adhesins FadA and Fap2 (10–12). Furthermore, *F. nucleatum* can stimulate tumor cells to secrete multiple cytokines and chemokines, including IL-8 and CXCL1, thus enhancing tumor growth and metastasis (13, 14).

Macrophages constitute a major component of the innate immune system response, exerting a broad spectrum of immunomodulatory effects and participating in the physiological processes of pathogen clearance, tissue homeostasis maintenance, and repair. Within the tumor microenvironment, macrophages typically display duality, differentiating into phenotypes with distinct functions: the pro-inflammatory M1 type and the anti-inflammatory, pro-tumor M2 type (15–17). Tumor-associated macrophages (TAMs), which predominantly exhibit M2 polarization, have been implicated in tumor progression, invasion, and metastasis (18, 19). Research has demonstrated that infection with *F. nucleatum* leads to an increased infiltration of TAMs at the tumor site, promoting M2 polarization and thereby accelerating colorectal cancer (CRC) progression (20–23). The regulation of macrophage polarization by *F. nucleatum* involves multiple signaling pathways, including NF-κB, STAT3, and PI3K/Akt (24–26). Hu et al. observed a significant abundance of M2-type tumor-infiltrating macrophages in CRC tissues positive for *F. nucleatum*, and *in vitro* macrophage polarization experiments corroborated that *F. nucleatum* induced M2 polarization. Their findings indicate that *F. nucleatum* promotes M2 macrophage polarization via activation of the TLR4/NF-κB/S100A9 cascade, thereby facilitating CRC progression (27). Xu et al. (28) reported that *F. nucleatum* enhanced CRC metastasis through the miR-1322/CCL20 axis and M2 polarization. Recent studies highlight the pivotal role of the NF-κB signaling pathway in macrophage polarization. Activation of the NF-κB pathway not only mediates the pro-inflammatory response of M1-type macrophages but also regulates the anti-inflammatory and reparative functions of M2-type macrophages (29, 30). M2-polarized macrophages contribute to tissue repair and remodeling of the tumor microenvironment by secreting anti-inflammatory cytokines such as IL-10 and TGF-β (18, 19). It has been reported that NF-κB p50 directly promotes the formation of M2-type macrophages by inhibiting the expression of pro-inflammatory gene and up-regulating the expression of M2 polarization-related genes. The NF-κB p100 may indirectly support M2 polarization by activating the non-classical NF-κB pathway (31).

Although previous research has indicated that the *F. nucleatum* is closely related to the occurrence and development of various inflammatory diseases and colorectal cancer. Compared with normal intestinal tissue, the enrichment of *F. nucleatum* is more significant in colorectal cancer tissue (9). In the field of colorectal cancer research, *F. nucleatum* is considered a key factor in tumor formation and development. Through its diverse pathogenic mechanisms, it plays an important role in the occurrence, development, immune evasion, and chemotherapy resistance of colorectal cancer. The components and metabolites of microorganisms can activate macrophages through specific pattern recognition receptors (PRRs) and guide them to differentiate into M1 or M2 types, thereby altering the characteristics of immune responses (32). *F. nucleatum* can bind with various immune cells such as macrophages and T cells, triggering immune suppression in the intestinal mucosa and creating a favorable microenvironment for the survival of colorectal cancer cells (33). In the tumor microenvironment, macrophages can differentiate into phenotypes with different functions, such as M1 type with pro-inflammatory functions and M2 type with anti-inflammatory and pro tumor functions (34). Regulating the polarization state of macrophages, especially inhibiting M2 or activating M1 macrophages, has become a potential strategy in tumor therapy. Therefore, studying the molecular mechanisms behind the polarization of macrophages into M1 or M2 not only deepens our understanding of the interaction between hosts and pathogens, but also provides a theoretical basis for exploring new therapeutic methods. This study explores and reveals the key molecular pathways by which *Fusobacterium nucleatum* infection induces M2 polarization in macrophages through transcriptomic analysis and experimental validation of the RAW264.7 infection model, in order to understand how *Fusobacterium nucleatum* promotes the development of colorectal cancer. In this study, we aim to elucidate the molecular mechanisms through which *F. nucleatum* infection induces M2 polarization of tumor-associated macrophages (TAMs) via the NF-κB pathway. The polarization direction of macrophages was characterized in a model of RAW264.7 macrophages infected with *F. nucleatum*. RNA-Seq transcriptome sequencing was performed on the transcriptional-level impaction of *F. nucleatum* infection. The investigation will deepen our understanding of the NF-κB pathway’s role in TAM polarization and potentially identify new therapeutic targets for CRC immunotherapy.

## 2 Materials and methods

### 2.1 Bacterial growth conditions

*F. nucleatum* ATCC 23726 was obtained from the American Type Culture Collection (ATCC) and routinely cultured in Tryptic Soy Broth (TSB) supplemented with 1% Bacto Peptone and 0.25% freshly prepared L-cysteine (TSPC). For solid cultures, TSPC agar plates were enriched with 1% Vitamin K1-hemin solution. All cultures were grown in an anaerobic incubator at 37°C under a gas mixture of 93% N_2_, 5% CO_2_, and 2% H_2_.

### 2.2 Infection model and invasion assay

RAW264.7 cells were seeded at a density of 1 × 10^6^ cells per 60-mm culture dish in 6 ml of complete DMEM (500 mL DMEM basic (1 ×) high glucose, 10% FBS Premium, 100U/mL Penicillin-Streptomycin Solution) and incubated at 37°C with 5% CO_2_ for 12 hours. Separately, *F. nucleatum* was cultured for 12 hours, collected by centrifugation (6,000 × g, 5 min, 4°C), and washed three times with PBS. The bacterial pellet was resuspended in DMEM (DMEM basic (1 ×) high glucose) to an OD_600_ of 1.0. Based on the desired multiplicity of infection (MOI), the appropriate volume of bacterial suspension was added to RAW264.7 cells for a 12-hour co-culture. After co-cultivation, cells were washed with PBS, and fresh DMEM (DMEM basic (1 ×) high glucose) was added. Cells were incubated for 1 hour to remove extracellular bacteria, followed by gentle washing with PBS. Intracellular *F. nucleatum* was released by lysing cells with cold sterile distilled water. Lysates were serially diluted and plated on TSPC agar for colony-forming unit (CFU) enumeration (35). Experiments were performed in triplicate.

### 2.3 Immunofluorescence analysis

Slides were pre-treated with detergent, rinsed with water, sterilized in 75% ethanol, and dried. RAW264.7 cells (1 × 10^6^) were seeded on slides following the infection protocol. After infection, cells were fixed with 4% paraformaldehyde for 10 -15 minutes and blocked with 3% BSA at room temperature for 30 minutes. Primary antibodies were added, and slides were incubated overnight at 4°C in a humidified chamber. After washing with PBS, fluorescent secondary antibodies were applied for 50 minutes at room temperature. DAPI staining was performed for 10 minutes in the dark. Slides were mounted with anti-fade medium, and fluorescence was visualized at excitation wavelengths of 330-380 nm (DAPI), 510-560 nm (Cy3-iNOS), and 465-495 nm (488-CD206). Images were acquired and analyzed.

### 2.4 Transcriptome analysis (RNA-Seq)

RAW264.7 cells were infected with *F. nucleatum* at an MOI of 100:1 for 4 hours. Total RNA was extracted using Trizol reagent, and three biological replicates were analyzed (36). Strand-specific RNA sequencing was conducted by Novogene using the Illumina HiSeq platform. Clean reads were mapped to the RAW264.7 genome using Bowtie2, and gene expression was quantified with HTSeq-count. Fragments per kilobase of transcript per million mapped reads (FPKM) values were calculated, with normalized data provided in Data Set S1.

### 2.5 RT-qPCR

Total RNA was extracted as described above. cDNA synthesis and quantification were performed using the BeyoFast™ SYBR Green One-Step qRT-PCR Kit (Beyotime) on a CFX96 Touch Real-Time PCR Detection System (Bio-Rad). GAPDH served as the reference gene. Each 20 µl reaction contained 500 ng RNA, 300 nM primers, 2 µl 10× SYBR Green One-Step Enzyme Mix, and 10 µl 2× SYBR Green One-Step Reaction Buffer. The primers used in this study are listed in **Table 1**. Reactions were conducted in triplicate.

**Table 1:** Primers used in this study.

Gene		Sequences
*GAPDH*	Forward primer	TCAACGGCACAGTCAAGG
	Reverse primer	ACTCCACGACATACTCAGC
*INOS*	Forward primer	CCCTTCCGAAGTTTCTGGCAGCAGC
	Reverse primer	CCAAAGCCACGAGGCTCTGACAGCC
*CD86*	Forward primer	TCAGTCAGGATGGGAGTGGTA
	Reverse primer	ATCCAAGAGCCATTCCTACCT
*CD206*	Forward primer	GTCATATCGGGTTGAGCCACT
	Reverse primer	AATCATTCCGTTCACCAGAGG
*MR*	Forward primer	CATGAGGCTTCTCTTGCTTCTG
	Reverse primer	TTGCCGTCTGAACTGAGATGG
16s	Forward primer	CAGAGTTTGATCCTGGCT
	Reverse primer	AGGAGGTGACCAGCCGCA
*Nfkb1*	Forward primer	GGCCTGCAAAGGTTATCGTT
	Reverse primer	CCGTGCTTCCAGTGTTTCAA
*Nkfb2*	Forward primer	CCAGAAACTTCAGAGGCAGC
	Reverse primer	TGGGAGATCACAGGCTTCAG
*Malt1*	Forward primer	AACCCAGAATCCAAGGCAGT
	Reverse primer	CTGTTGTTAACCCGGCAGAC
*Lta*	Forward primer	GAGCAACAACTCCCTCCTGA
	Reverse primer	GAGGCACATGGAAGGGGTAT
*Ltb*	Forward primer	TCACCCTCTAGCCTCTCAGA
	Reverse primer	GTTGAACCCCTGGATCTGGT
*Nfkbia*	Forward primer	TTGGTCAGGTGAAGGGAGAC
	Reverse primer	CAGGCAAGATGTAGAGGGGT
*Tnf*	Forward primer	TGCTTGTTCCTCAGCCTCTT
	Reverse primer	AGATGATCTGACTGCCTGGG

### 2.6 Western blotting

RAW264.7 cells infected with *F. nucleatum* were washed with cold PBS and lysed in RIPA buffer containing protease and phosphatase inhibitors. Lysates were centrifuged (12,000 × g, 15 min, 4°C), and protein concentrations were measured using the BCA assay. Equal protein amounts (20-30 µg) were separated by SDS-PAGE, transferred to PVDF membranes, and blocked with 5% non-fat milk in TBS-T for 1 hour. Membranes were incubated with primary antibodies targeting P50, P100, and GAPDH overnight at 4°C, followed by HRP-conjugated secondary antibodies. Protein bands were visualized using ECL and quantified with ImageJ. Experiments were performed in triplicate.

### 2.7 Scanning electron microscopy

Log-phase *F. nucleatum* cultures were centrifuged (6,000 × g, 5 min, 4°C) and fixed in 2.5% glutaraldehyde at 4°C for 4 hours. Samples were dehydrated in ethanol gradients (25%, 50%, 75%, 100%) and air-dried on silicon chips. Dried samples were gold-coated and visualized under SEM. RAW264.7 cells underwent a similar fixation, dehydration, and coating process before SEM observation.

### 2.8 Statistical analyses

Data are expressed as mean ± SD. Statistical differences were determined using unpaired Student’s t-test or one-way ANOVA with Bonferroni *post hoc* analysis. Differences were considered significant at p < 0.05. Analyses were performed using GraphPad Prism. Significance levels are indicated as follows: p < 0.05, *p < 0.01, **p < 0.001, a and not significant (N.S., p > 0.05).

## 3 Results

### 3.1 *F. nucleatum* infection promotes M2 macrophage polarization

**Figure 1:**
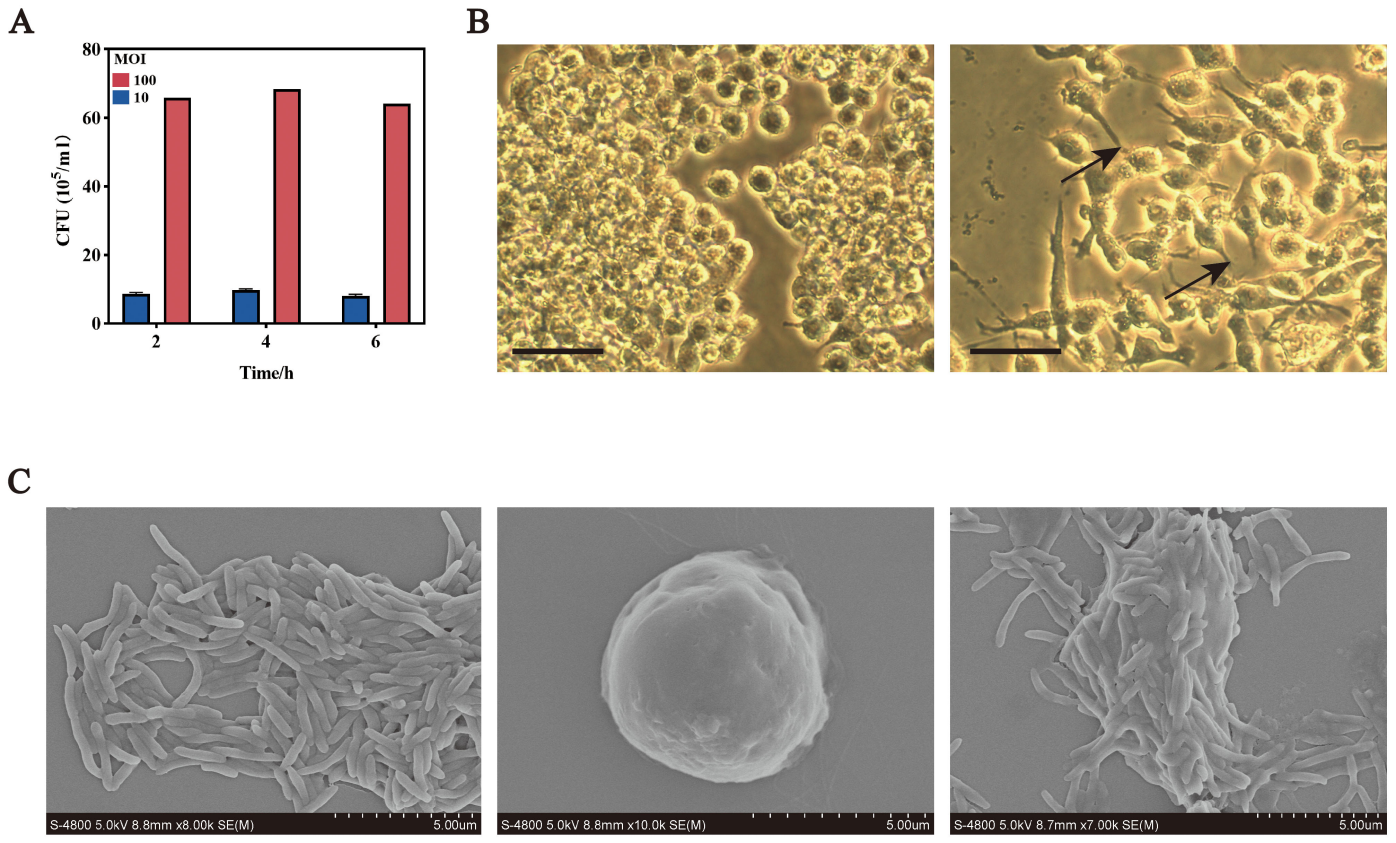
*F. nucleatum* infection promotes M2 macrophage polarization. **(A)** Intracellular bacterial counts after *F. nucleatum* infection at different MOIs over time. **(B)** Optical microscopy images of RAW264.7 cells in normal (left) and polarized states (right) bars, 10 um. **(C)** Scanning electron microscopy images of *F. nucleatum* and RAW264.7 cells. Left, *F. nucleatum*; middle, RAW264.7; right, RAW264.7 after *F. nucleatum* infection; Bars, 5 um.

To investigate the effect of *F. nucleatum* on macrophage polarization, RAW264.7 cells were infected at MOIs of 10:1 and 100:1. CFU measurements over 6 hours revealed that at MOI 10:1, bacterial counts peaked at 4 hours (1.0 × 10^6^/ml) and decreased by 6 hours (9.0 × 10^5^/ml). At MOI 100:1, counts similarly peaked at 4 hours (7.0 × 10^6^/ml). Therefore, MOI 100:1 with a 4-hour infection period was chosen for subsequent experiments (**Figure 1A**).

Microscopic analyses revealed morphological changes in infected RAW264.7 cells. Optical microscopy showed transformation from round to polarized shapes with projections (**Figure 1B**), while scanning electron microscopy confirmed elongated forms with spike-like projections and bacterial adhesion to the cell surface (**Figure 1C**).

**Figure 2:**
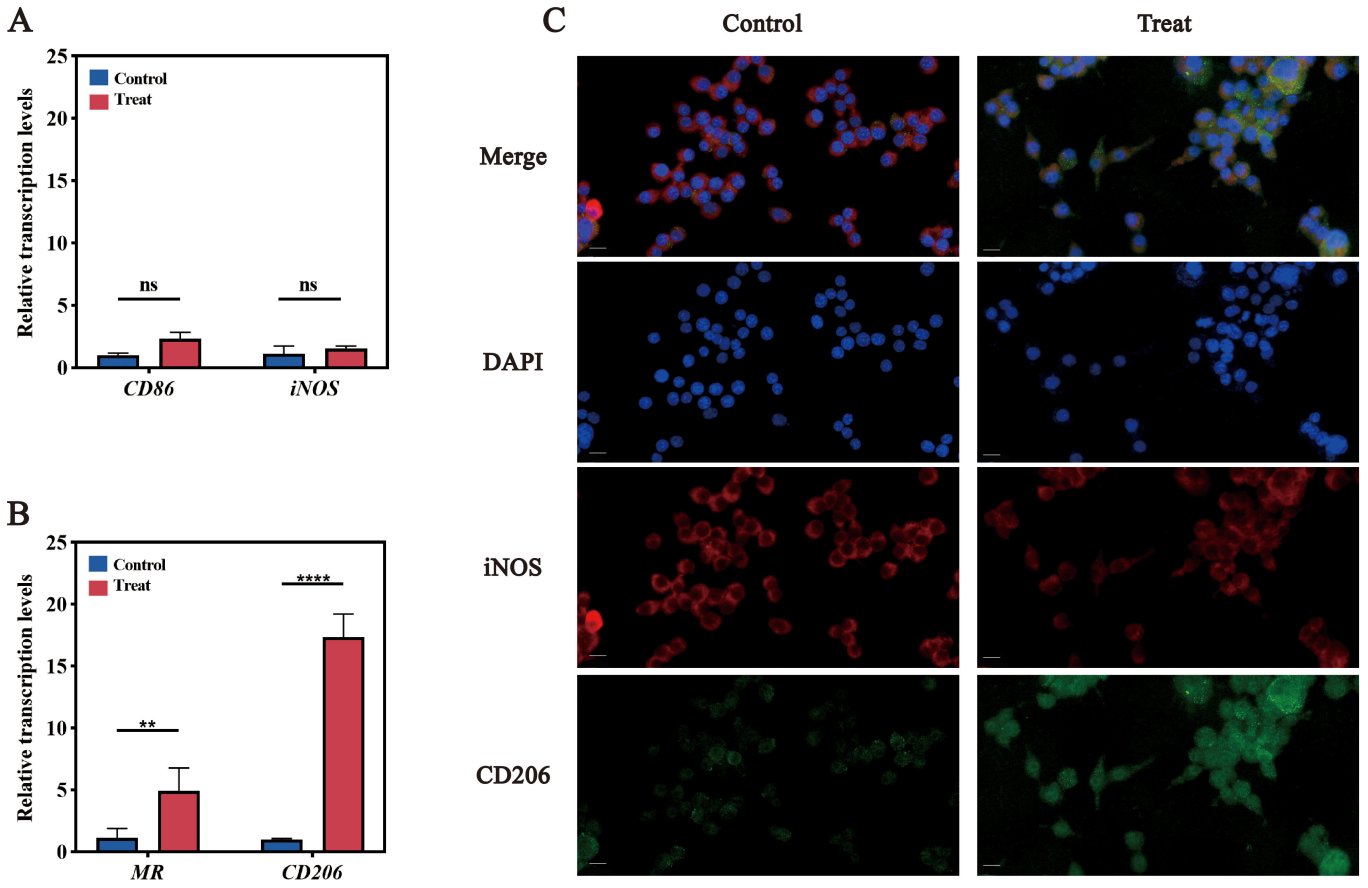
Validation of M2 polarization in RAW264.7 cells. **(A)** qPCR results for M1 polarization marker genes, CD86 and iNOS. **(B)** qPCR results for M2 polarization marker genes, MR and CD206. **(C)** Immunofluorescence images of M1 and M2 polarization. DAPI stains nuclei in blue; iNOS (red) is the marker for M1 polarization, and CD206 (green) is the marker for M2 polarization. ns (not significant): P > 0.05, indicating no statistically significant difference.

RT-qPCR analysis demonstrated significant upregulation of M2 markers (C*d206* and *Mr*) following infection, with minimal changes in M1 markers (*Cd86* and *Nos2*) (**Figures 2A, B**). Immunofluorescence analysis supported these findings: CD206 protein expression increased, while iNOS fluorescence intensity decreased (**Figure 2C**). These results indicate that *F. nucleatum* infection drives M2 polarization in RAW264.7 macrophages.

### 3.2 Differential gene expression analysis

**Figure 3:**
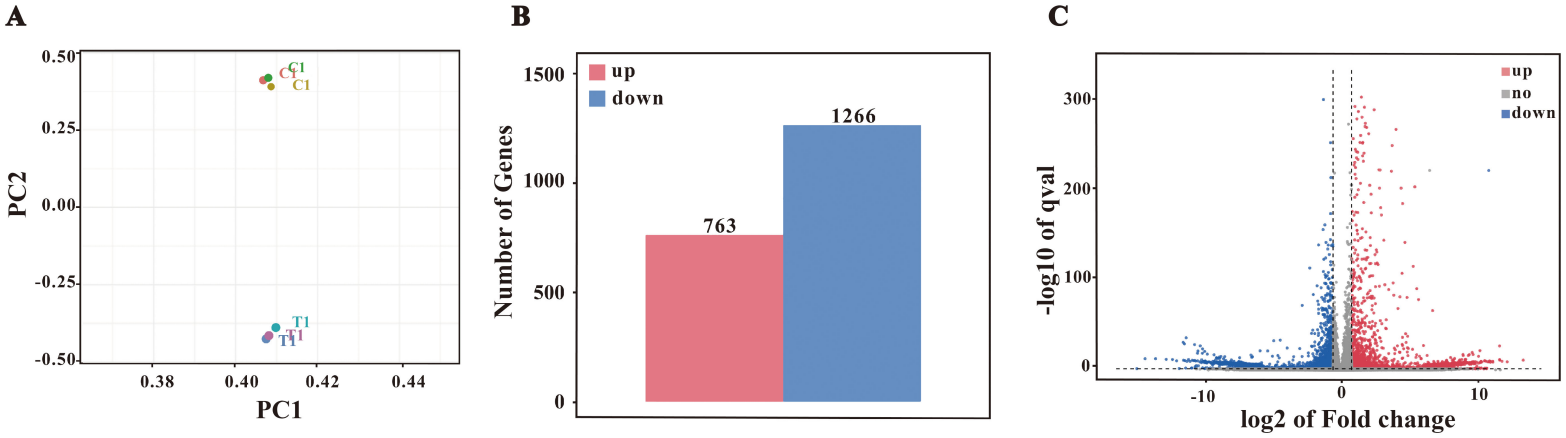
Differential gene expression analysis. **(A)** PCA plot. **(B)** Bar chart of differentially expressed genes (DEGs). Differential expression analysis identified 2029 DEGs, with 763 upregulated and 1266 downregulated genes. **(C)** Volcano plot of DEGs.

Transcriptomic profiling was performed to explore the molecular mechanisms underlying *F. nucleatum*-induced M2 polarization. Sequencing generated high-quality data (Q20/Q30> 97%; GC content: 49.0-49.5%), and PCA confirmed distinct clustering between control and infected samples (**Figure 3A**). Using a threshold of Fold Change ≥ 2 or ≤ 0.5 (i.e., |Fold Change| ≥ 2) and a q-value < 0.05, a total of 2,029 differentially expressed genes were identified, comprising 763 upregulated and 1,266 downregulated genes (**Figure 3B**).

Based on the volcano plot analysis (**Figure 3C**), we identified significant upregulation of several genes, including *Il12b*, *H2-Ea*, *Nox3*, *Ptgs2*, and *Muc3*, which suggests that *F. nucleatum* infection may induce macrophage polarization toward the M2 phenotype. While *Il12b* is typically associated with Th1 responses, its upregulation may contribute to M2 polarization through immune regulatory pathways under certain conditions. The increased expression of *H2-Ea*, an antigen presentation-related gene, aligns with the tissue repair role of M2 macrophages. Additionally, the upregulation of *Ptgs2* indicates that COX-2 and its product PGE2 may promote M2 polarization through anti-inflammatory mechanisms, while the expression of *Nox3* could be linked to the metabolic reprogramming required for M2 polarization. Furthermore, enhanced expression of *Muc3* suggests that macrophages may support host defense by strengthening barrier functions.

These gene expression changes are consistent with our findings from fluorescent immunostaining and RT-qPCR experiments, providing additional evidence that *F. nucleatum* infection drives M2 polarization in macrophages. Overall, these results highlight a potential link between *F. nucleatum* infection and M2 macrophage polarization, offering a foundation for further investigation into the underlying molecular mechanisms.

### 3.3 Functional enrichment analysis

**Figure 4:**
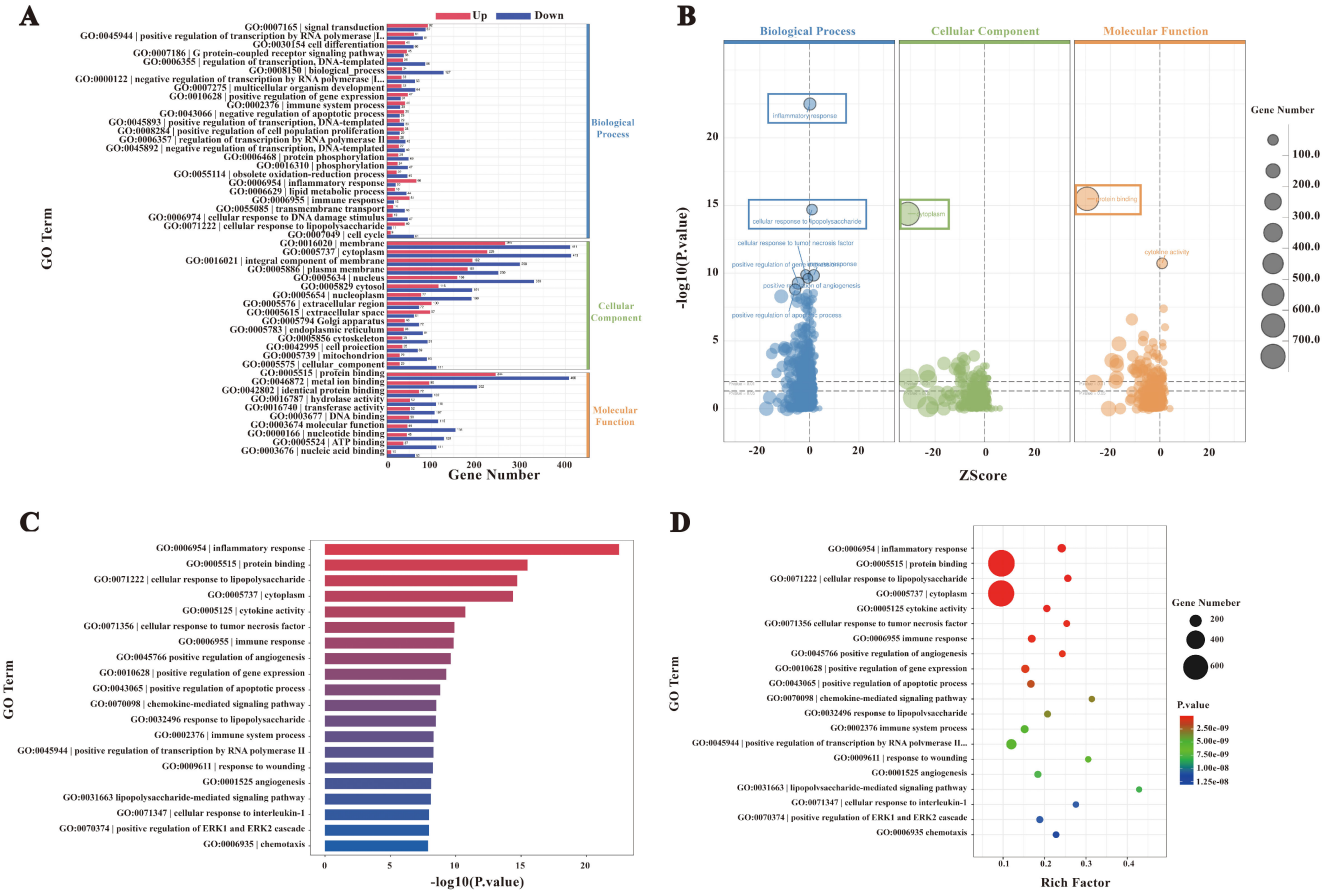
GO enrichment analysis. **(A)** Bar chart of GO enrichment statistics for DEGs. **(B)** GO enrichment bubble plot. **(C)** Bar chart of the top 20 GO terms with the lowest p-values. **(D)** GO enrichment scatter plot.

A total of 2,099 differentially expressed genes (DEGs) were subjected to Gene Ontology (GO) enrichment analysis, with results summarized in **Figure 4A**. The DEGs were categorized into biological processes (BP, 34%, 2,074 genes), cellular components (CC, 50%, 4,347 genes), and molecular functions (MF, 26%, 2,203 genes). These distributions reflect the extensive alterations in cellular functions and components induced by *F. nucleatum* infection. In the BP category, significantly enriched terms included immune-related processes such as inflammatory response, cellular response to lipopolysaccharide, and cellular response to tumor necrosis factor. In the CC category, DEGs were predominantly localized to the cytoplasm, indicating substantial changes in intracellular dynamics post-infection. Enriched terms in the MF category, such as protein binding and cytokine activity, suggested enhanced protein interactions and the secretion of signaling molecules (**Figure 4B**).

The top 20 GO terms with the lowest P-values, visualized in a bar chart (**Figure 4C**), showed significant enrichment in processes such as inflammatory response, cytokine activity, and cellular response to chemical stimuli. These terms underscore the extensive transcriptional reprogramming of macrophages following infection, involving immune activation, signal transduction, and metabolic adaptation. Furthermore, a bubble plot analysis highlighted dynamic changes in genes linked to inflammation, immune regulation, and metabolic processes (**Figure 4D**). Collectively, these findings indicate that *F. nucleatum* infection induces profound transcriptional alterations in macrophages, enhancing protein interactions and activating immune signaling networks to reinforce host defenses against pathogenic invasion.

**Figure 5:**
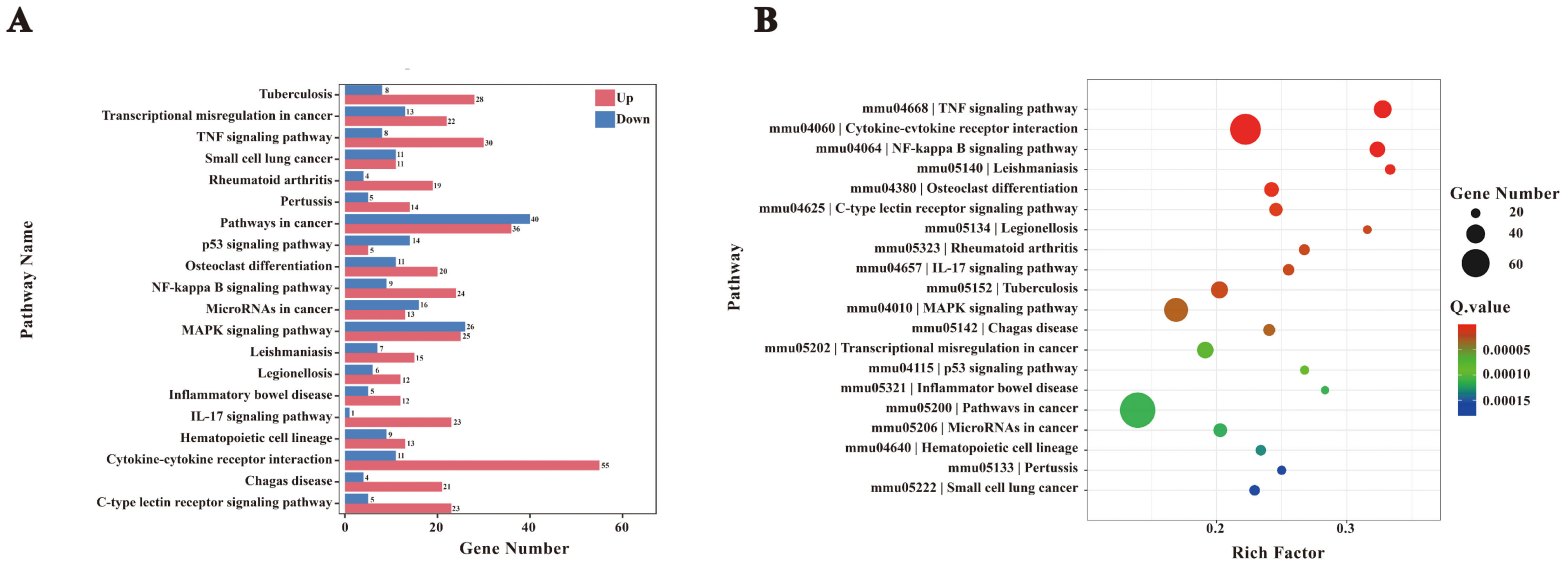
KEGG enrichment analysis. **(A)** Bar chart of KEGG enrichment statistics for DEGs. **(B)** KEGG enrichment bubble plot. To interpret KEGG enrichment pathways from multiple perspectives, the bubble plot was constructed. The x-axis represents the enrichment factor, and the y-axis shows pathway names. The size of the bubbles represents the number of significantly enriched genes annotated to each GO term, while the color indicates the Q value.

The biological significance of these molecular functions was further validated through KEGG enrichment analysis, which identified the NF-κB signaling pathway as a critical mediator of the host immune response during *F. nucleatum* infection (**Figure 5**). KEGG analysis also revealed that cytokine-cytokine receptor interactions and NF-κB activation play key roles in orchestrating immune and inflammatory responses. Specifically, NF-κB signaling drives M2 polarization of macrophages by coordinating cytokine-mediated interactions, influencing disease progression and host-pathogen dynamics.

### 3.4 Protein-protein interaction and NF-κB pathway analysis

**Figure 6:**
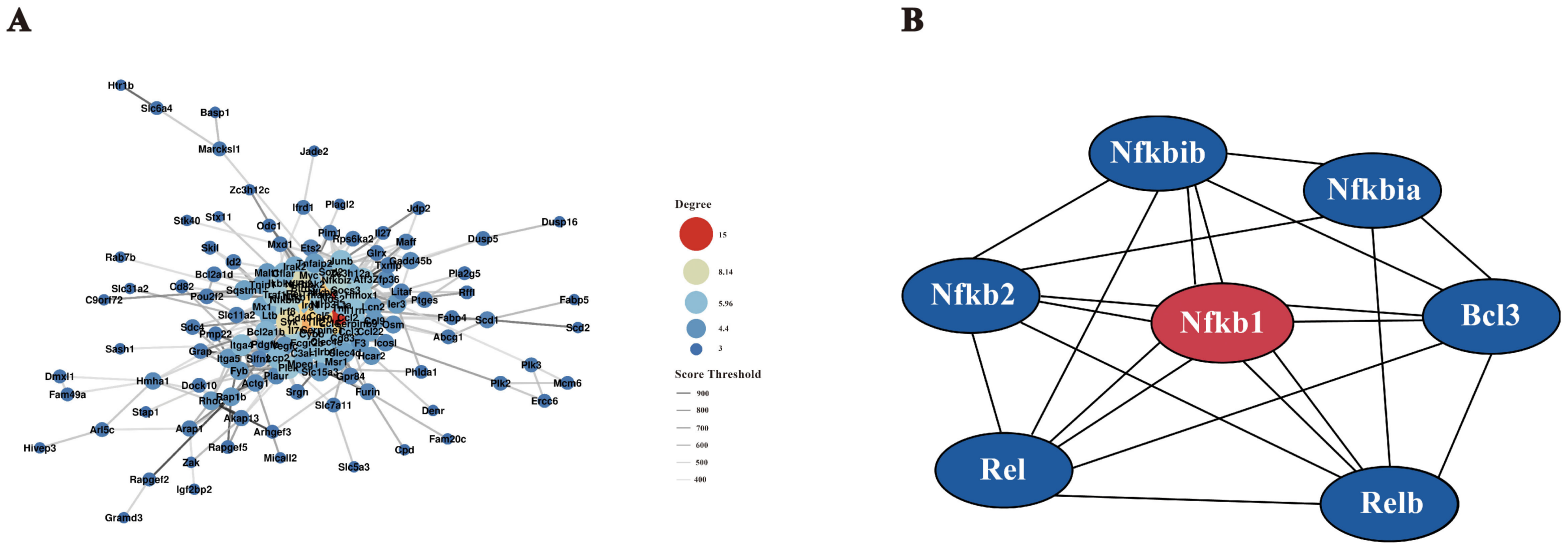
Interaction network diagram. **(A)** Protein-protein interaction (PPI) network. Using the STRING protein interaction database, interaction networks of all DEGs were analyzed, filtering for interaction scores >400. The PPI network was visualized using Cytoscape software. **(B)** NF-κB gene interaction network. The STRING database was used to analyze interactions between key NF-κB pathway proteins and DEGs from transcriptome analysis. The network was visualized using Cytoscape software.

Protein-protein interactions among DEGs (score>400) were analyzed using the STRING database, and the resulting interaction network was visualized with Cytoscape (**Figure 6A**). Key genes with high connectivity, including *Ccl2*, *Ccl5*, *Ccl4* (involved in immune cell recruitment), *Tnf*, and *Il10* (regulators of inflammation), were identified in **Table 2**. Notably, *Tnf* emerged as a central hub within the NF-κB pathway, influencing cell survival, proliferation, and inflammation modulation. These findings suggest a complex regulatory network that modulates macrophage immune responses upon *F. nucleatum* infection. Within the NF-κB pathway, regulatory genes such as *Nfkbia* and *Nfkb1* play pivotal roles in macrophage polarization and inflammatory responses. *Nfkbia* controls the duration of NF-κB activity, while *Nfkb1* drives the expression of inflammatory genes. *Tnf*, a key cytokine, amplifies NF-κB signaling and promotes M2 polarization by enhancing tissue repair and anti-inflammatory functions mediated by cytokines like IL-10 and TGF-β. These interactions support the hypothesis that *F. nucleatum* infection induces M2 polarization through the TNF/NF-κB axis.

**Table 2:** Gene node number.

Gene	Nodes	Description of function	References
Tumor necrosis factor (*Tnf*)	78	A principal inflammatory cytokine that modulates inflammatory and immune responses	(37).
Monocyte chemotactic protein 2 (*Ccl2*)	53	A chemokine functions to recruit and activate monocytes into the area of inflammation	(38, 39)
Interleukin-10 (*Il10*)	52	A pivotal anti-inflammatory cytokine.	(40).
toll-like receptor 2, Squamous cell carcinoma associated protein 2 (*Tlr2*)	51	Playing a crucial role in the activation of the innate immune system through the recognition of pathogen-associated molecular patterns	(41).
Nuclear factor kappa B repressor alpha regulates NF - κ B signaling (*Nfkbia*)	45	Participates in various immune and inflammatory responses	(42)
CD40 molecule (*Cd40*)	41	A crucial receptor in the immune system that plays a key role in modulating B cell proliferation and antibody production	(43)
RANTES (*Ccl5*)	40	Potentially contributing to the recruitment of immune cells to the site of inflammation	(44)
Nuclear factor kappa B1 (*Nfkb1*)	40	A pleiotropic transcription factor implicated in the regulation of inflammatory and immune responses	(45)
MIP-1 β (*Ccl4*)	40	A chemokine that plays a crucial role in the recruitment and activation of immune cells	(38, 39)
Spleen tyrosine kinase (*Syk*)	36	Regulatory molecules involved in the signal transduction pathways of B cell receptors and other immune receptors	(46)

To elucidate gene interactions within the NF-κB pathway, a gene interaction network was constructed using Cytoscape (**Figure 6B**). Nodes represent individual genes, while edges denote interactions. *Nfkb1* emerged as the central node with extensive connections, highlighting its crucial role in regulating immune responses, inflammation, and cell fate. Other key genes, including *Rel, Relb, Nfkbia, Nfkibb, Nfkb2*, and *Bcl3*, were identified as interacting partners. For instance, *Nfkbia* and *Nfkibb* inhibit *Nfkb1* activity by forming inhibitory complexes, which can be reversed upon specific signals, allowing *Nfkb1* to translocate into the nucleus and regulate target gene expression. Additionally, *Bcl3* modulates NF-κB activity by binding to the complex, thereby fine-tuning downstream responses. These findings provide a comprehensive view of the NF-κB-mediated regulatory network during *F. nucleatum* infection, revealing its role in promoting M2 macrophage polarization and coordinating immune responses to infection.

### 3.5 Validation of NF-κB pathway activation

Transcriptomic analysis identified a clear upregulation of the NF-κB signaling pathway in RAW264.7 macrophages following *F. nucleatum* infection. To validate this observation and elucidate the molecular mechanisms underlying M2 macrophage polarization, we conducted RT-qPCR and Western Blotting to assess NF-κB pathway activation at both mRNA and protein levels. These experiments aimed to verify the hypothesis that *F. nucleatum* drives M2 polarization through TNF/NF-κB pathway activation.

**Figure 7:**
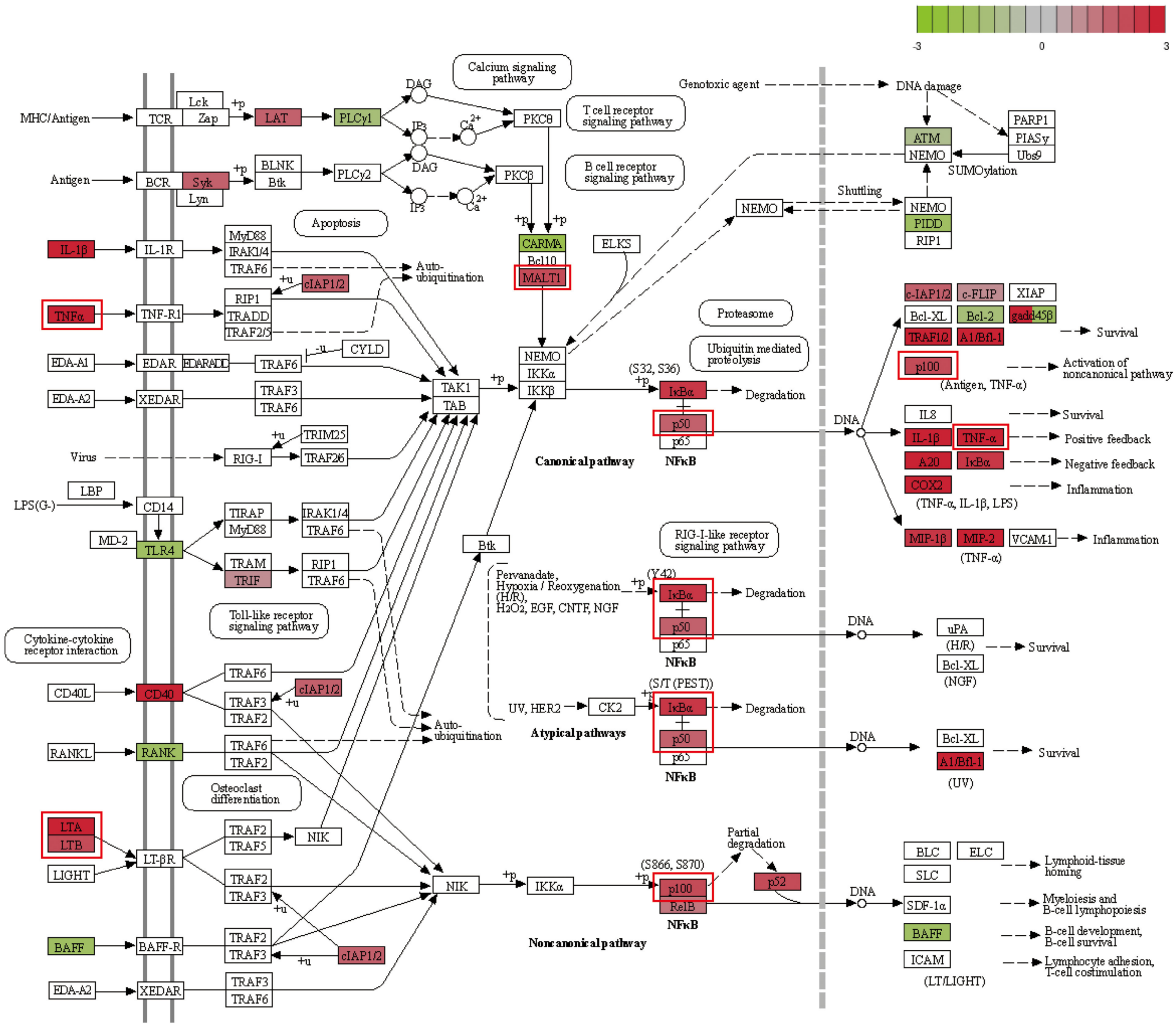
NF-κB pathway map. Upregulated genes are marked in red, with deeper colors indicating higher log2(fold change). Downregulated genes are marked in green, with deeper colors indicating lower log2(fold change).

Transcriptomic data revealed significant upregulation of key NF-κB pathway genes, including *Il1b*, *Tnf*, *Cd40*, *Nfkbia*, *Nfkb1*, *Traf1*, and *Traf2*, while genes such as *Tnfsf13b*, *Rank*, and *Tlr4* were downregulated (**Figure 7**). Upregulated genes were highlighted in red and downregulated genes in green, with intensity reflecting their respective log2 fold changes. Among these, the central role of *Nfkb1* in the interaction network underscored its importance in regulating immune responses, inflammation, and macrophage polarization. Previous researches demonstrated that *Nfkb1*, along with other pathway components such as *Nfkbia*, *Rel*, and *Relb*, forms dynamic complexes to modulate gene expression in response to infection stimuli.

RT-qPCR analysis confirmed the significant upregulation of *Tnf*, *Malt1*, *Nfkb1*, and *Nfkb2* following infection, consistent with transcriptomic findings. Elevated expression of MALT1 and TNF, two critical mediators of NF-κB signaling, reflects enhanced cellular responses to infection, potentially amplifying immune and inflammatory processes in RAW264.7 cells. Additionally, tumor necrosis factor-related genes *Lta* and *Ltb* were upregulated, suggesting a pro-inflammatory response facilitated by NF-κB pathway activity.

**Figure 8:**
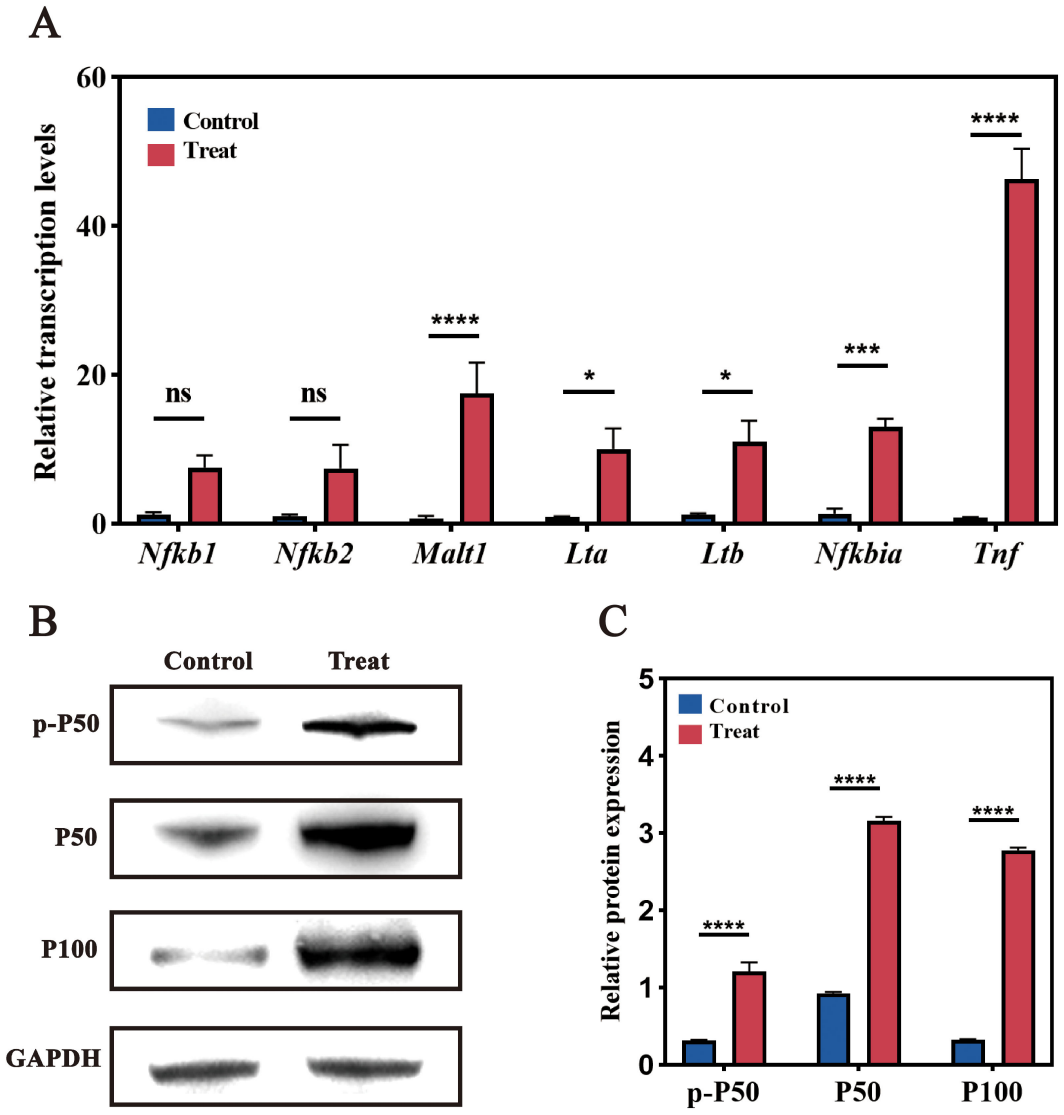
Validation of NF-κB pathway-related genes. **(A)** RT-qPCR analysis of NF-κB pathway-related genes. Significant changes in expression levels were observed post-infection compared to the uninfected control group. **(B)** Western blot analysis. Bands show protein expression in Control and Treat (post-infection) samples. Increased expression of P50 and P100 was observed in infected cells compared to the control group. **(C)** Quantitative analysis of Western blot band intensity. ns (not significant): P > 0.05, indicating no statistically significant difference. *: P≤0.05, indicating a statistically significant difference. **: P≤0.01, indicating a very significant difference. ***: P≤0.001, indicating an extremely significant difference. ****: P≤0.0001, indicating an exceedingly significant difference.

Western blotting further corroborated these findings, revealing increased expression of NF-κB pathway proteins P50 and P100 in infected cells compared to controls (**Figure 8**). Quantitative analysis of band intensities confirmed a significant rise in P50 and P100 protein levels, validating NF-κB activation at the protein level. This upregulation aligns with RT-qPCR results, indicating enhanced immune and inflammatory responses in RAW264.7 cells under *F. nucleatum*-induced stress.

Together, these results provide compelling evidence that the NF-κB pathway serves as a central regulator in the macrophage response to *F. nucleatum* infection. The observed activation of NF-κB signaling corroborates transcriptomic data, supporting the hypothesis that *F. nucleatum* induces M2 macrophage polarization through TNF/NF-κB signaling, thereby modulating host defense and inflammatory responses.

## 4 Discussion

In this study, we systematically examined the impact of *F. nucleatum* infection on macrophage polarization, particularly the mechanism by which it promotes M2 polarization of RAW264.7 cells via the NF-κB signaling pathway. By establishing an *in vitro* infection model, we demonstrate that *F. nucleatum* infection not only induces morphological alterations in macrophages but also significantly upregulates M2 polarization-associated markers (CD206, MR). The infection of macrophages by *F. nucleatum* has little effect on cell proliferation (The relevant experimental results are not presented in this article.). Our findings align with and expand upon previous studies, highlighting the complex interplay between *F. nucleatum* and macrophage polarization in CRC (20, 27). These results offer novel mechanistic insights into the role of *F. nucleatum* in promoting immune regulation within the tumor microenvironment.

Through the application of scanning electron microscopy, RT-qPCR, and immunofluorescence assays, it was observed that RAW264.7 cells exhibited characteristic M2-type polarization following infection with *F. nucleatum*. This finding is consistent with other reports that *F. nucleatum* promotes M2 polarization of macrophages in the microenvironment of CRC. By promoting an M2 phenotype in macrophages, *F. nucleatum* appears to suppress anti-tumor immunity and foster a microenvironment conducive to tumor progression (20, 47–49). Similar effects have been observed in other cancer types, such as oral squamous cell carcinoma. For example, *F. nucleatum* activates NF-κB signaling to promote an inflammatory and immunosuppressive TME (50). Notably, our study observed morphological changes in macrophages post-infection, along with upregulation of key NF-κB pathway genes (*Nfkb1, Nfkb2, Tnf*, and *Malt1*), further establishing *F. nucleatum*’s contribution to an immunosuppressive TME in CRC.

Similar to *F. nucleatum*, other pathogens such as *Helicobacter pylori* and pathogenic *E. coli* strains have been shown to influence macrophage polarization and contribute to tumor progression through immune modulation. For instance, *H. pylori*, a well-known gastric cancer risk factor, drives M2 polarization via the cag pathogenicity island and NF-κB activation, promoting immune tolerance within the gastric TME (51). Similarly, *E. coli* strains harboring pks islands can induce DNA damage in colon cells and drive M2 macrophage polarization, contributing to CRC development by creating an immunosuppressive environment (52). Another example is P*orphyromonas gingivalis*, an oral pathogen implicated in pancreatic cancer, which activates NF-κB signaling to induce M2 polarization and foster a tumor-supportive immune profile (53).

These examples underscore a shared strategy among pathogens, wherein NF-κB activation leads to M2 macrophage polarization, enabling immune evasion and facilitating tumor progression (54, 55). While NF-κB signaling is a key driver of the M2-polarizing effects of pathogens like *H. pylori* and *E. coli*, our study uniquely highlights *F. nucleatum*’s role in CRC. Beyond inducing M2 polarization, *F. nucleatum* upregulates genes involved in angiogenesis, cytokine signaling, and immune responses, underscoring its multifaceted regulation of the TME and its significant contribution to CRC progression.

Through RNA-Seq and GSEA analysis, we identified that *F. nucleatum* infection markedly upregulates several signaling pathways associated with inflammation and tumor progression, notably key genes in the NF-κB pathway, including *Nfkb1*, *Nfkb2*, *Tnf*, and *Malt1*. Subsequent RT-qPCR and Western Blot analyses confirmed a significant increase in the expression levels of P50 and P100 proteins following infection, which suggests that the NF-κB pathway is crucial in regulating M2-type polarization induced by *F. nucleatus* infection. These fundings suggest that disrupting the M2 macrophage phenotype or inhibiting NF-κB signaling may offer new strategies to combat pathogen-driven CRC. T These strategies may also be broadly applicable to other cancers associated with chronic bacterial infections, such as those linked to *H. pylori*, *E. coli*, and *P. gingivalis*. Collectively, this study lays the groundwork for developing pathogen-targeted immunotherapies that could transform the management of CRC and other infection-associated cancers.

This study provides a strong experimental basis for *F. nucleatum*-induced M2 macrophage polarization but has limitations. It uses *in vitro* cell models without *in vivo* validation. Future research should use mouse models to strengthen the link between *F. nucleatum* infection and M2 polarization, improving clinical relevance. Additionally, validating the association in colorectal cancer patient samples could assess *F. nucleatum*’s potential as a biomarker or therapeutic target.

## 5 Conclusion

In conclusion, this study demonstrates that *F. nucleatum* induces M2 macrophage polarization through NF-κB activation, contributing to an immunosuppressive tumor microenvironment in colorectal cancer (CRC). By comparing *F. nucleatum* with other pathogens utilizing similar mechanisms, we highlight the broader role of bacterial modulation of macrophages in cancer progression. These findings improve our understanding of *F. nucleatum*’s involvement in CRC and may guide the development of immunomodulatory therapies targeting macrophage reprogramming or NF-κB pathways, potentially enhancing outcomes in pathogen-associated cancers.

## Data Availability

The datasets presented in this study can be found in online repositories. The names of the repository/repositories and accession number(s) can be found in the article/supplementary material.
